# Improving Chinese College Students’ Argumentative Writing: A Presentation-Assimilation-Discussion-Exercise Approach

**DOI:** 10.3389/fpsyg.2022.874531

**Published:** 2022-06-29

**Authors:** Menglin Liao, Yuanxi Liao

**Affiliations:** ^1^Department of Foreign Languages, Hainan Medical University, Haikou, China; ^2^Development and Reform Research Center, Hainan Normal University, Haikou, China

**Keywords:** PADE model, argumentative writing, writing quality, argumentative structure, evaluation

## Abstract

This study implemented the Presentation-Assimilation-Discussion-Exercise (PADE) model, a student-centered teaching model that originated in China, and examined its effect on college students’ argumentative writing. Quantitative method was used in this study following a teaching practice of 14 weeks. A total of 76 Chinese first-year university students of intermediate English level with 38 students in an experimental class and 38 students in a comparison class took part in the study. Students from the experimental class received the PADE model, and the comparison class received traditional teaching. Students from both classes were asked to compose two argumentative essays before and after the treatment. At the end of the treatment, students completed questionnaires on the PADE teaching model. Students’ writings were evaluated on aspects of linguistic quality and argumentative structure. The results indicated that students who learned in the PADE teaching environment outperformed students who followed traditional teaching method in the post-writing, and significant differences were shown in all aspects except organization and grammar. The questionnaire finding suggested that students from the experimental class held a welcoming attitude toward the PADE model and benefited from it from the perspectives of course design, teaching arrangement, and learning effect. The PADE teaching has implications for teaching writing in contexts that share many similarities.

## Introduction

Writing plays a pivotal role in the learners’ foreign language development and meanwhile is commonly perceived as more difficult than the other three language skills, namely, listening, speaking, and reading. Argumentative writing is challenging since it requires students to have knowledge of the sentence structure, format, and content of the argument (Pratiwi, 2016). A foreign language is more of an issue for student writers as they are obliged to remain sophisticated in language use and persuasive in the delivery of viewpoints. Individual’s understanding and application in argumentation were difficult to improve without sustained instructional focus and require multifaceted aspects of argumentation knowledge (Ryu and Sandoval, 2012; Kuhn et al., 2013). Therefore, it comes as no surprise that many strategies have been proposed in the field of foreign language education in order to improve the foreign language learners’ argumentative writing skills, which, however, seem only a bit successful. This is because these strategies tend to pay much attention to the effect of instruction in argumentative writing (VanDerHeide, 2018). Argumentation is also a social negotiation that involves knowledge construction and critique through negotiation (Chen et al., 2016b). Therefore, students need to understand an argument epistemologically with social negotiation to develop their argumentation. Social writing is important to refine and consolidate the new ideas (Rivard and Straw, 2000). However, interaction during the writing process is, to a large degree, ignored by the instructors. In addition, though researchers realized the importance of knowledge construction in writing argumentation, only a few empirical studies adopted applicable teaching model in secondary education (Andrews et al., 2006; Jeffers, 2018), let alone its implementations in tertiary education. Thus, argumentative writing intervention programs for tertiary education associating both knowledge construction plus social practice are needed.

For the purpose of introducing more interactions in English as a foreign language (EFL) writing teaching and promoting students’ argumentative writing competence, this study introduces the Presentation-Assimilation-Discussion-Exercise (PADE) model as an alternative way to improve the learners’ argumentative writing skills in tertiary education. This PADE model was based on Zhang’s (2014) Presentation-Assimilation-Discussion (PAD) model, which had three main sessions originally. In comparison to the existing writing teaching strategies, the PADE model combines argumentative writing learning in association with knowledge construction at early stages and social interaction and writing skill practice at later stages. Each stage of the PADE paves the way for the next, and they compose a virtuous cycle of learning, self-studying, understanding, and mastering. To investigate the effectiveness of the PADE model in enhancing the college students’ argumentative writing, pre-writing and post-writing performance of an experimental class and a controlled class were compared and analyzed from the aspect of argumentative structure and overall writing quality. In addition, the experimental class students’ perception of the teaching model was collected by questionnaires. The findings of the study could offer new insights for teaching English to foreign language learners of varied English levels.

## Literature Review

### Argumentative Writing

Toulmin (1958) first proposed the concept of argumentation. Kuhn (1991) considered argumentation an essential thinking skill for idea formulation, problem-solving, and good judgment. In early literature, argumentation was defined as a genre to deal with a real or imagined difference of an opinion on a controversial issue (Van Eemeren et al., 2019). As an important genre of writing, argumentation aims to persuade readers with clarified claims and adequate supporting evidence. Toulmin et al. (1990) created an argumentative model, including claim, grounds, warrant, backing, qualifier, and rebuttal, and it had been used widely on various subjects. Also, it was proved to be effective to improve argumentation skills, reflective thinking, and academic performance. For the purpose to construct a high-quality argumentation, on the one hand, in argumentation, writers have to use their knowledge of argumentative discourse, topic, and critical standards of evaluation to present and evaluate their writing (Ferretti and De La Paz, 2011; Ferretti and Graham, 2019). On the other hand, various skills should be associated with the argumentative writing task, such as organizing skills, problem-solving, critical thinking, and knowledge construction. High requirements on writers’ grammatical competence and discourse competence also need to be met.

As a complex and advanced writing task, many native speakers find it not easy to construct a convincing argumentation. Hence, it poses a greater challenge for foreign language learners. Compared with the expert writers, novices are especially poor at possessing enough genre and topic knowledge, thus having great difficulty regulating their writing process (Harris et al., 2011; McCutchen, 2011; Graham et al., 2013). The development of argumentation requires students’ understanding of the multifaceted aspects of argumentation (Chen et al., 2016a). Many students lack training, thus having great language difficulties, such as using cohesive devices and distinguishing oral and written language and correct use of tenses, articles, and preposition (Liu and Braine, 2005; Fareed et al., 2016). In addition to the language problems, EFL learners have cultural barriers and different thinking patterns that may hinder their reasoning in argumentative writing and also have great difficulties in using scientific evidence to support justifications, analyzing and critiquing arguments, and making justified claims and recognizing opposing arguments (Sadler, 2004). Arsyad (1999) found that Indonesian EFL college students usually did not use counterarguments in argumentation which may be deemed as impolite, especially for people of higher social ranks. Another reason for foreign language learners’ weakness in argumentation could be attributed to poor argumentative writing skills and structure (Liu and Stapleton, 2014). For most Chinese college students, although they have always been good at listening and reading skills, they performed relatively weak in writing, especially in argumentative writing. Their main problems in argumentative writing include limited vocabulary, word repetition, wrong sentence structure, and grammar weakness (Liu and Li, 2017).

### Intervention Programs for Argumentative Writing

To solve EFL students’ struggles in argumentation writing, researchers have designed intervention programs for argumentative writing from the perspective of instructional strategies and argumentative structure (Stern and Solomon, 2006). Explicit instruction and writing workshops were proved to be effective as pedagogy to improve students’ writing. With explicit instructions of the instructor, students were able to use more linguistic resources to analyze after the teaching practice (Pessoa et al., 2019). Ong and Zhang (2010) increased the task complexity of EFL students and explored its effect on fluency and lexical complexity of argumentative writing. Lacum et al. (2014) asked students to read research papers with special attention to the rhetorical moves in the authors’ arguments. After the reading, students’ argumentation was more closely related to the topic with better choice of words and elaboration. Argumentative structure is another important element that helps to organize writing in a well-presented way. Effect of prior knowledge in improving students’ formulation of claims and reasons was examined (Wolfe et al., 2009). A similar study conducted by Voss (2005) found that students learned from argumentative schemata to organize claims, reasons, warrants, and counterarguments in their argumentative writing. A second-order argument scaffold was proposed and practical guidelines were provided to ensure the acquisition and application of the argumentation skills with support of students’ internal argumentative script and other external computer-supported tools (Noroozi et al., 2018). Computer-Supported Argumentative Writer (C-SAW), an online software, was employed in argumentative writing and proved to be effective in generating and elaborating arguments; its visual schema also helped students to integrate knowledge about argumentative writing components (Benetos and Bétrancourt, 2020).

Argumentative writing is regarded as not only a simple writing process that requires explicit instruction and social interaction; communication also plays important role in further developing argumentative structure. Well-argued ideas, for example, arguments and debates bring people and their ideas into contact and make sense of new ideas and experience collaboratively in disagreement (Newell et al., 2011). Through peer interactions and communication, students fostered and inspired their thinking, thus, their argumentation was more logical and organized. Researchers have exemplified the role of peer interaction in argumentative writing. Providing feedback proved to be essential to find out various problems in writing so as to correct students’ errors, and was helpful to improve students’ argumentation (Ryandini, 2019). Lin et al. (2012) examined the effect of reflective asynchronous discussions on quality and complexity of college students’ argumentation. It revealed that the asynchronous online communication group outperformed its counterparts in all of the three argumentation topics. Specifically, the asynchronous online communication group created more rebuttals than the paper–pencil group. Blended learning, which is associated with offline and online collaborative argumentation tasks, was used to help students to deal with their language problems, as well as improved arguments (Jin et al., 2020). Tavakoli and Rezazadeh (2014) explored the individuals and collaboratively planned conditions on fluency, complexity, and accuracy of Iranian EFL learners’ argumentative writing performance, and the result indicated that the collaboratively planned condition improved more accurate argumentation. Zioga and Bikos (2020) adopted a collaborative writing program through the Google Docs writing tool and found that students improved significantly in nearly all the structural elements of argumentative discourse. The findings confirmed the effect of cooperative learning in argumentative writing. Alternative teaching methods of supportive reciprocal interactions (Lunsford, 2002), classroom talk moves (VanDerHeide, 2018), and patterns of talk and write (Chen et al., 2016b) were also employed to facilitate argument.

### The Presentation-Assimilation-Discussion-Exercise Model in Argumentative Writing

Although various teaching interventions were used to improve argumentative writing, most of the previous studies did not associate argumentative writing teaching with students’ interactions. In that case, learners could not reach a further stage to fully understand the knowledge and were able to apply and transfer them into argumentation. Therefore, empirical argumentative writing teaching program for the EFL context that associates both argumentative writing knowledge and skill input with sufficient interaction is needed.

Zhang (2014) in China first proposed the PAD teaching model based on cognitive psychology theory, which inspired students to participate actively in the learning process. Many Chinese researchers have introduced the PAD model in English teaching in tertiary education; however, most of them only focused on how to design the PAD model teaching scaffold for English class without further empirical practice (Sun, 2016; Wang and Huang, 2016). Almost no PAD study focused on English writing in a specific genre.

Taking advantage of knowledge presentation, skills input, and peer interactions, this study modified the PAD model and designed a PADE teaching model. This study aimed to establish a learning circle that consolidated students’ writing performance step by step. Exercise (E) stage was added to the original PAD model to reinforce and practice students’ understanding of writing skills so as to improve students’ writing performance during the process. Argumentation is regarded as a series of language practices from the language perspective (Klein, 2006). With sufficient writing practices, students’ writing skills as well as way of thinking are stimulated (Kartawijaya, 2018). The teaching model referenced previous writing interventions and combined knowledge construction with large amounts of social practice. It enabled students to have sufficient knowledge input at presentation (P) and assimilation (A) stage to improve their grammar and linguistic competence. It is worth noticing that the discussion session (D) offered students the opportunity in class to develop concepts and solve their problems through peer interaction and improved their writing during the process of generating ideas, evaluating, and justifying (Chen et al., 2013). In this study, the PADE argumentative writing teaching model was conducted among 76 non-English major college students and questionnaires were designed and sent in the experimental class. It aimed at exploring the impact of the PADE teaching model on college students’ argumentative essay and indicated whether it improved college students’ linguistic competence and argumentative structure in their argumentative writing. Comparisons and analysis were made to investigate the differences between two classes and their development in argumentation after receiving the PADE teaching and traditional treatment. The experimental class students’ perception of the PADE teaching model was collected by questionnaires. The specific research questions of this study are as follows:

1. Does the PADE model in college English improve students’ argumentative writing in terms of vocabulary, grammar, organization, content, argumentative structure, and overall quality?
2. What do students perceive about the PADE approach to English argumentative writing?

## Materials and Methods

The main purpose of conducting this study was to explore the effect of the PADE model on college English argumentative writing. Quantitative data between experimental and comparison class students’ argumentative essays were collected and analyzed from the perspective of overall linguistic quality and argumentative structure. Students’ perceptions of the PADE model in college English argumentative writing were also gathered.

### Participants

This study conducted teaching practices in an experimental class and a control class in a university in a medium-sized capital city in the southern province of China. A total of 76 first-year non-English major students in an experimental class and a comparison class with intermediate English level participated in the teaching practice. The students’ age ranged from 18 to 20. There were 38 students in the experimental class (14 females and 24 males) and 38 students in the comparison class (16 females and 22 males). Students in the experimental and comparison class were of similar intermediate English proficiency. Their final English performance in first semester of first year showed no significant differences. The two classes were taught by the same teacher to ensure the flow of teaching plan. In the second semester of the first year, the experimental class received the PADE treatment and the comparison class followed traditional way of English teaching. Before the teaching practice, both classes first received 2 weeks’ trial training of the PADE model and traditional model to ensure that they were familiar with the flow of the PADE model and traditional way of English teaching. After being acquainted with the teaching design of the PADE model and traditional teaching, the researchers began to conduct the teaching practice.

### Experimental Design

The teaching practice was designed to answer the research questions. To respond to the first question, whether students in the experimental class followed the PADE model in college English argumentative writing outperformed students in the comparison class on perspective of vocabulary, grammar, organization, content, argumentative structure, and overall quality of argumentative essay, teacher assigned two argumentations of equal difficulties of National College English Test Band 4 (CET 4) following the direction of CET 4 before and after the treatment. Before the treatment, students from both classes were required to submit an argumentative essay on the topic – *Should we take liberal arts courses?* – on Pigai,^1^ an automated writing evaluation website. Then, at the end of the teaching practice, students were asked to hand in another argumentative essay – *Should we go shopping online?* – on Pigai website. The online writing environment enabled students to submit their essays with instructional support and peer feedback, which facilitated higher writing quality (Noroozi et al., 2011; Latifi et al., 2021). Students were required to write within the time limit of 60 min with no less than 120 words after the class.

To answer the second question, questionnaires on students’ perception of the PADE teaching model were designed and then distributed to the experimental class to learn students’ attitudes toward the PADE teaching model.

### Instructional Procedures for Experimental Class

**FIGURE 1:**
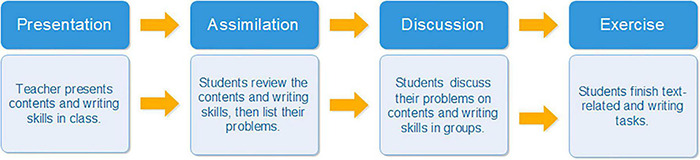
The implementation of the PADE argumentative writing teaching practice.

The PADE model in English argumentative teaching of each unit took 2 weeks. Each followed four phases of the PADE (Figure 1). In the first week, teachers emphasized content of unit to construct linguistic knowledge for the experimental class. Text content, as well as linguistic focuses, was made clear by teachers during the presentation session. After the presentation, students in the experimental class reviewed the knowledge they learned and listed their problems by themselves after the class. In the discussion session, students in the experimental class discussed their problems in groups for the purpose of solving their puzzles as well as achieving a better understanding of the content through peer communication and sharing. At the end of the unit teaching, teachers assigned text-related exercises to students to finish and consolidate students’ knowledge of language and content.

The second week’s teaching focused on argumentative writing skills. Teachers first presented the basics of argumentative writing skills with evaluation rubric in the experimental class. After the class, students in the experimental class listed their problems in understanding their argumentative writing skills. In the discussion class, students in the experimental class discussed in groups to further understand the argumentative writing skills and their key elements. During the discussion, students gave and received feedback from each other, which was effective in the learning and writing process and outcomes. They learnt to evaluate peers’ argumentative essay objectively based on the writing rubric (Huisman et al., 2018; Noroozi, 2018). At the end of the class, students were asked to practice their argumentative writing skills after the class (Table 1).

**TABLE 1:** The implementation of the PADE and traditional teaching model for one unit.

Week	Session	Experimental class	Comparison class
Week 1	In class	Presentation: Teacher presents content and linguistic knowledge of the unit.	Presentation: Teacher presents content and linguistic knowledge of the unit and assigns homework.
	After class	Assimilation: Students gather text-related materials and list their problems.	Exercise: Finish text-related exercises after class.
	In class	Discussion: Students discuss their problems in groups in class.	Presentation: Teacher checks the answers to the exercise in class.
	After class	Exercise: Finish text-related exercises after class.	
Week 2	In class	Presentation: Teacher introduces the writing skills and rubric of the unit.	Presentation: Teacher introduces the writing skills and rubric and assigns homework.
	After class	Assimilation: Students list their problems with writing skills.	Exercise: Finish writing skills activities.
	In class	Discussion: Students discuss their problems in groups and in class.	Presentation: Teacher reviews the writing skills.
	After class	Exercise: Finish writing skills activities.	
Week 3			
…			

### Instructional Procedures for Controlled Class

For the comparison class, teaching basically is consisted of presentation and exercises every week. In the first class of the first week, teachers presented the content of unit in class with linguistic focuses and assigned the text exercises. In the second class, teachers checked the answers of the exercise in class. In the second week, argumentative writing skills, along with writing rubric, were presented by teachers with assignment. Students finished the writing skills tasks in the fourth class (Table 1).

### Instruments

#### Writing Performance Evaluation

College English Test is a nationally standardized test that evaluates college students’ English competence objectively and accurately. This study assigned argumentative writing tasks with CET 4 difficulty, which is suitable for students with intermediate English level. Students from both classes were required to submit their argumentation of the required topics with similar CET 4 direction. Students were required to write individually on the writing website Pigai, which could automatically evaluate students’ essays with prompt feedback and was used widely in Chinese universities. Upon finishing the essay, Pigai graded students’ writing with a specific score based on CET 4 grading metric set by the researchers. Essays with high duplicate check rate were required to submit again. By researching main scoring rubrics for tests of ESL writing, vocabulary, grammar, content, and organization were set as the main indicators of argumentation quality combined with Pigai writing system rubrics. In addition, argumentative structures were judged by the most frequently used Osborne et al. (2004) rubric with the lowest level 1 to highest level 5.

#### The Learners’ Perception Questionnaire

Questionnaire was used to understand students’ perception of the PADE model on the argumentative teaching. It was composed of four parts in a total of 15 items, namely, the PADE course design (5 items), the PADE teaching effect (3 items), the PADE learning effects (5 items), and implication of the PADE teaching into other courses (2 items). Other survey addressed students’ engagement was used as supplement to the questionnaire (Kühnen et al., 2012; Liao and Li, 2017). The whole research team and experienced professors in the English Department checked the questionnaire. Based on the experts’ feedback, the researcher modified the questionnaire. The questionnaires’ validity was high (Kaiser–Meyer–Olkin value was 0.809 and Bartlett’s test of sphericity value was 0.000). The Cronbach’s alpha of the questionnaire achieved 0.927, which verified the good reliability of the questionnaire. A 5-point Likert was used in the questionnaire (1 = strongly disagree and 5 = strongly agree). Instructors explained the direction of questionnaires in detail at the beginning and then asked students to finish items according to their real situation in class within 20 min. Printed copies of questionnaires were sent to students a week before the end of the teaching practice to investigate students’ perceptions of the PADE model in English argumentative writing teaching.

#### Data Collection and Analysis

As this study focused on argumentative writing, researchers employed Osborne et al. (2004) argumentation rubric, along with Hedgcock and Lefkowitz’s (1992) writing rubric, to holistically grade students’ argumentation (Appendix Tables A1,A2). The essays’ overall linguistic quality was judged from the perspective of vocabulary, grammar, organization, and content with reference to Hedgcock and Lefkowitz (1992) rubrics. Each part was composed of a 0–100 scale. Osborne et al. (2004) argumentation structural rubric that includes levels 1–5 was employed. Level 1 is the lowest level, which includes a simple claim or counterclaim without supporting details. Level 3 has claims with data, warrants, and backings. Level 5 ranks the highest, which incorporates more than one rebuttal in the argument.

To examine discrepancy between two classes, two researchers with equivalent teaching and research experience scored vocabulary, grammar, organization, content, argumentative structure, and overall quality of essays written before and after the teaching practice with reference to Pigai website’s automated assessment. Once two teachers had disagreement and had two score’s rating difference on linguistic quality and 1 level difference on argumentative level, an expert was invited to reevaluate the essay. The average score of the two teachers was taken as students’ final performance. The interrater reliability between the two scorers on linguistic quality and argumentative structure was 0.826 and 0.813, respectively.

Quantitative research method was adopted in this study. The social software SPSS 22.0 was used to analyze the data. Descriptive analysis was conducted first to show the holistic picture of two class performance. To answer question 1, paired-samples *t*-tests were used to present the argumentation development of the two classes during the intervention. ANCOVA was conducted to explore difference between the two classes’ post-writing after the teaching intervention. Assumption tests were conducted for the paired-samples *t*-test and ANCOVA. The skewness and kurtosis values showed that normality assumption was met for the paired-samples *t*-test and ANCOVA. The simple scatter plots were conducted and the patterns of lines were displayed, resembling the linearity of ANCOVA. In addition, the homogeneity assumption was run using Levene’s test. The result showed that the homogeneity met the requirement of ANCOVA.

The questionnaire was designed focused on students’ perception of the PADE treatment’s effect on students’ English writing. To answer question 2, descriptive data of each item in the questionnaire were shown to illustrate students’ perception toward the PADE teaching model. Percentages on each item were calculated. Mean score and standard deviation of each subscale were generated.

## Results

### Students’ Argumentation Development

To get a general picture of the two classes’ essay performance, descriptive analysis was conducted first. The two classes achieved similar scores on item of vocabulary in their first essay; after the teaching practice, students of the experimental class improved their vocabulary performance by 5.91. However, students from the comparison class almost kept the same performance. Another noticeable improvement was in the aspect of content. Both classes had no significant differences on their first essay’s content performance. However, the experimental class students’ score on their second essay climbed to 93.02 while the comparison class students’ performance on content of their second essay only had a minor improvement of 0.47. The students from the experimental class maintained their advantage in grammar and organization. In general, the experimental class students and their counterparts performed equivalently in the first essay but the gap between them widened in the second essay on vocabulary, content, and overall quality, followed by slight advantages of organization and grammar. As for argumentative structure, students from the experimental class were above the midst of levels 3 and 4 after the training while students from the comparison class scored just above level 3 after the training.

The paired-samples *t*-tests showed that within the teaching intervention, the experimental class improved greatly on their vocabulary, organization, content, and argumentative structure except for grammar. Following the traditional writing teaching method, students from the controlled class also improved their writing quality and argumentative structure. However, their advancement was minor compared to the experimental class and had no significant development in the vocabulary, grammar, organization, and content (Table 2).

**TABLE 2:** Descriptive and paired-samples *t*-test results of the two classes’ essay performance.

	Experimental class						Comparison class					
	Pairing	*N*	*M*	SD	*t*	*p*	Pairing	*N*	*M*	SD	*t*	*p*
Vocabulary	1	38	75.53	4.27	−3.511	0.001	1	38	75.17	8.14	−0.165	0.870
	2	38	81.44	8.56			2	38	75.43	8.69		
Grammar	1	38	74.91	9.00	−0.365	0.717	1	38	74.79	8.37	−0.315	0.755
	2	38	75.56	5.47			2	38	75.42	9.20		
Organization	1	38	72.23	5.71	−3.650	0.001	1	38	72.44	8.58	−1.056	0.298
	2	38	76.36	5.21			2	38	74.71	9.60		
Content	1	38	78.12	17.70	−4.161	0.000	1	38	76.33	8.10	−0.266	0.792
	2	38	93.02	9.05			2	38	76.80	8.65		
Overall quality	1	38	75.53	6.69	−5.750	0.000	1	38	75.27	5.57	−3.184	0.003
	2	38	83.24	4.35			2	38	79.28	3.79		
Argumentative structure	1	38	3.18	0.39	−4.275	0.000	1	38	2.89	0.31	−2.458	0.019
	2	38	3.63	0.54			2	38	3.11	0.45		

A one-way ANCOVA was used to examine the effect of the PADE teaching model on students’ argumentation. Students’ essays written before the teaching practice were taken as a covariate to find out their influence on the post-writing on aspect of vocabulary, grammar, organization, content, overall writing quality, as well as argumentative structure. The difference between the two classes’ post-writing was also examined. The post-writing performance was taken as a dependent variable. The experimental class and comparison class were taken as between-group factors while time performed as the within-subjects factor (pre-writing and post-writing).

A significant group difference was shown by one-way ANCOVA analysis in the post-writing between the two classes, and the Bonferroni test revealed that the experimental student performed significantly better on vocabulary than their counterparts [*F*(1,73) = 9.033, *p* = 0.040, η_*p*_^2^ = 0.110]. The difference between two classes’ writing in terms of grammar was not significant [*F*(1,73 = 0.007), *p* = 0.932, η_*p*_^2^ = 0.000]. Students from the experimental class performed significantly better on post-writing’s content [*F*(1,73) = 65.251, *p* = 0.000, η_*p*_^2^ = 0.472]. However, there was no significant difference in the post-writing’s organization [*F*(1,73) = 0.852, *p* = 0.359, η_*p*_^2^ = 0.012]. For the average performance, the between-group effect analysis revealed a significant difference and the students from the experimental class scored significantly higher than those from the controlled class [*F*(1,73) = 18.684, *p* = 0.000, η_*p*_^2^ = 0.204)]. To evaluate students’ argumentative structure specifically, the result showed that the experimental class significantly outperformed the controlled class on argumentative structure [*F*(1,73) = 15.852, *p* = 0.000, η_*p*_^2^ = 0.178] (Table 3).

**TABLE 3:** One-way ANCOVA results of the two classes’ essay performance.

	Groups	*N*	Pre-writing *M* (SD)	Post-writing *M* (SD)	*F*	*p*	η_*p*_^2^
Vocabulary	Experimental class	38	75.53 (4.27)	81.44 (8.56)	9.033	0.040	0.110
	Controlled class	38	75.17 (8.14)	75.43 (8.69)			
Grammar	Experimental class	38	74.91 (9.00)	75.56 (5.47)	0.007	0.932	0.000
	Controlled class	38	74.79 (8.37)	75.42 (9.20)			
Organization	Experimental class	38	72.23 (5.71)	76.36 (5.21)	0.852	0.359	0.012
	Controlled class	38	72.44 (8.58)	74.71 (9.60)			
Content	Experimental class	38	78.12 (17.70)	93.02 (9.05)	65.251	0.000	0.472
	Controlled class	38	76.33 (8.10)	76.80 (8.65)			
Overall quality	Experimental class	38	75.53 (6.69)	83.24 (4.35)	18.684	0.000	0.204
	Controlled class	38	75.27 (5.57)	79.28 (3.79)			
Argumentative structure	Experimental class	38	3.18 (0.39)	3.63 (0.54)	15.852	0.000	0.178
	Controlled class	38	2.89 (0.31)	3.11 (0.45)			

### Students’ Perceptions of the Presentation-Assimilation-Discussion-Exercise Model

#### The Presentation-Assimilation-Discussion-Exercise Course Design

**FIGURE 2:**
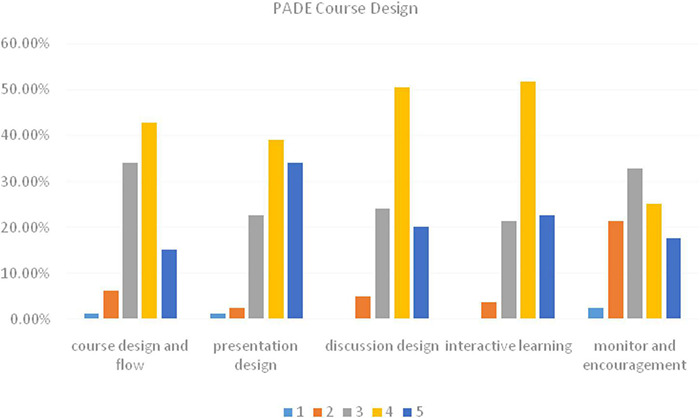
Percentage of the PADE course design: 1 – strongly disagree; 2 – disagree; 3 – neutral; 4 – agree; 5 – strongly agree.

Students were well-accepting of the PADE model in general (Figure 2). Concerning the teaching design and flow of the PADE teaching, 43% of students agreed with the new teaching model. Nearly 15.2% of students were strongly satisfied with the PADE teaching sessions featured with teaching contents with appropriate difficulty and clarified teaching sessions. Presentation helped students to understand the difficulties and laid a solid foundation for after-class understanding and further discussion session. Notably, 39.2% of students were satisfied with presentation session of the PADE model, and 34.2% of students were highly satisfied with it. About half of the students agreed with the core session of discussion. During the group discussion, students solved each other’s problems, thus facilitating individual’s understanding of the teaching content. Only about 5.1% of students disapproved of that item. About 75% of students were satisfied with the design of the PADE model and considered that it has created a better interactive learning environment that improved students’ English learning. The PADE course design urged and monitored each student to further study and understand the content in time; therefore, students learned the lesson step by step and solved their difficulties promptly. Also, 43% of students agreed with the item while 2.5% of students strongly disagreed with it. For the whole part, most students were satisfied with the design of the PADE model in college English argumentation teaching, with a high satisfaction rate (*M* = 3.64, SD = 0.84).

#### The Presentation-Assimilation-Discussion-Exercise Teaching Effect

**FIGURE 3:**
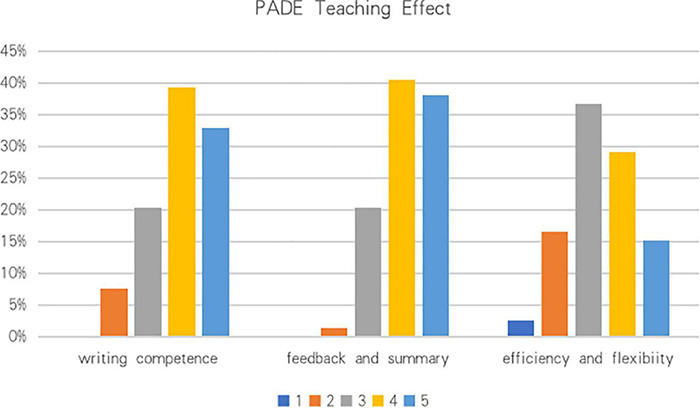
Percentage of the PADE teaching effect: 1 – strongly disagree; 2 – disagree; 3 – neutral; 4 – agree; 5 – strongly agree.

Over 72% of students viewed the PADE model as an effective teaching model to improve their general English argumentative writing competence (Figure 3). In the traditional large-size teacher-centered class, teachers usually find it extremely hard to monitor each student’s study and solve their problems in time. In the PADE teaching, an overwhelming majority (78.5%) of students were satisfied with their learning for timely feedback and summary provided by teachers and peers. In the PADE class, teachers could monitor individual students’ English study progress with great efficiency. Most of the students were happy with the efficiency and flexibility of the PADE teaching, which also cultivated their English interest and self-discipline, and only 2.5% of students strongly disapproved of the great efficiency of the PADE model. In general, students were pleased with teachers’ teaching in the PADE model (*M* = 3.83, SD = 0.91).

### Learning Effect of the Presentation-Assimilation-Discussion-Exercise Model

**FIGURE 4:**
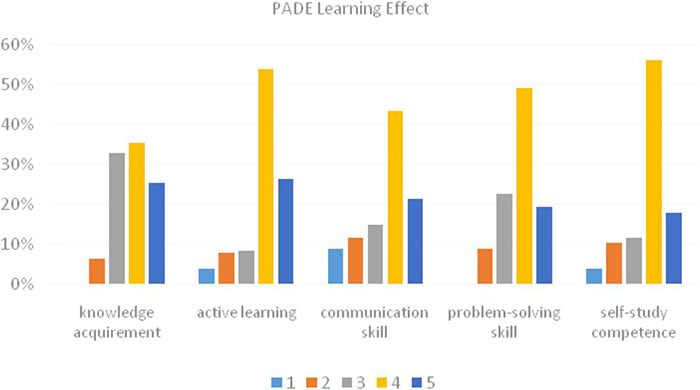
Percentage of the PADE learning effect: 1 – strongly disagree; 2 – disagree; 3 – neutral; 4 – agree; 5 – strongly agree.

The finding indicated that students were satisfied with their learning effect of the PADE model in argumentation writing (Figure 4). Although a small number of students (6.3%) expressed that they learned little knowledge in the PADE model, 60.7% of students were satisfied with the PADE model in achieving a better understanding of the teaching content. Compared with traditional teaching, students were pushed to think critically about the contents that benefited their argumentative writing greatly. Nearly 80% of students were in favor of the teaching practice from the perspective of encouraging their active thinking. In the discussion session, students were required to communicate with group members in English to discuss their problems in English writing learning. Around 65% of students indicated that they improved their communication skill and transferred what they discussed into writing. Furthermore, in the PADE learning, students had to study by themselves after the class to better understand the content and found out their own problems, which fostered their self-regulation and ensure the smooth progress of discussion. After the discussion session, nearly 70% of students admitted that their problems had been solved. Only 3.8% of students indicated that they had no improvement in their self-study competence, but most of the students admitted that they had cultivated self-study ability during the PADE teaching practice, which also benefited other discipline. In summary, students were satisfied with their learning effect of the PADE model (*M* = 3.19, SD = 0.82).

### Application of the Presentation-Assimilation-Discussion-Exercise Model to Other Courses

**FIGURE 5:**
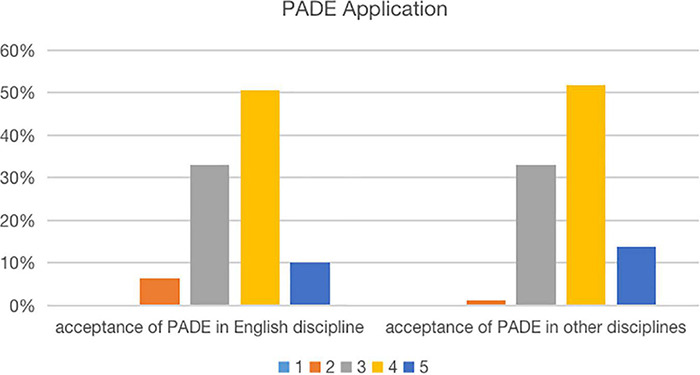
Percentage of the PADE application: 1 – strongly disagree; 2 – disagree; 3 – neutral; 4 – agree; 5 – strongly agree.

After the teaching practice of the PADE model, 60.7% of students were supportive of the use of the PADE model in learning English courses. Over half of the students (65.6%) were in favor of the implication of the PADE model to other courses (Figure 5). The finding indicated that students were in well-acceptation of the PADE model (*M* = 3.07, SD = 0.67).

## Discussion

This study conducted the PADE and traditional ways of teaching in English argumentation on participants of intermediate English level in an experimental and a comparison class. It compared and analyzed argumentative writings of the two classes written before and after the argumentation teaching practice for the purpose of exploring the impact of the PADE teaching model on students’ argumentative essay quality and argumentative structure. Students’ perception of the PADE model was also analyzed. The students from the experimental class improved their argumentation within the treatment on all aspects except for grammar. The PADE teaching method significantly influenced both classes’ post-writing on all aspects but organization and grammar. The reason might be attributable to the lack of emphasize on grammar during the practice. In addition, the students of intermediate English level from both classes had difficulties to improve grammar within the period. This is also in line with Chang and Lu’s (2018) study, which used a three-step prewriting activity but found no significant difference in organization and argumentation between two groups’ students. The result proved the effect of the PADE treatment in teaching argumentation with empirical findings. The finding from the questionnaires indicated that students were well-accepted of the PADE teaching model in the aspect of course design, teaching effect and learning effect, and they were willing to apply it to other courses in the future.

### The Presentation-Assimilation-Discussion-Exercise Model’s Effects on Argumentation Quality

By observing the essays, students from the experimental class presented more specific and professional vocabulary in their writing. The reason might be attributed to the effect of group discussions on L2 reading comprehension (Turnbull and Evans, 2017). With the help of group discussion session of the PADE treatment, students learnt to use specific words for communicative purpose, thus they expressed in a clear and logical way so that other group members could understand their claims, grounds, and backings clearly. During the process, they also learnt from others and enlarged their vocabulary. Thanks to more opportunity to express themselves in class, students from the experimental class became more adept in expressing their opinions well in the oral discussion and were able to transfer their thinking in argumentative writing. Apart from that, the PADE’s presentation session also offered students sufficient time to review the new words and expressions they learnt.

Additionally, the PADE model proved to be effective in improving students’ organization in writing. The reason might be the positive influence of preparation and presentation for discussion. To prepare for the discussion session, the experimental students were encouraged to think critically to find out their problems during language learning. Argumentation not only developed students’ epistemic knowledge, but also enhanced their critical thinking skills (Chen et al., 2020). In discussion, students cultivated critical thinking, organized their thinking in logic, and then presented their ideas clearly later within groups. With the discussion and exercise sessions, students’ organization and overall writing quality were gradually enhanced. In contrast, teachers found out students’ common problems with language and argumentative structure within the session of discussion, thus, addressing them with prompt feedback efficiently. This is in alignment with Chen’s (2019) finding on collaborative writing that the experimental class performed significantly better on vocabulary and organization in immediate post-tests.

Significant difference in the content of the second argumentation was shown between the two classes. The PADE model first input substantial linguistic knowledge in presentation, the later discussion and exercise session further consolidated the knowledge student learnt through interactions and practices. Hence, students understood the writing skills better and were able to relate them in argumentative writing. As a high-demanding writing task, argumentative writing obliged students to produce complex ideas in well organization, and the peer feedback during the discussion session improved students’ content learning and writing performance (Rahimi, 2019; Noroozi et al., 2020). The study indicated that students from the experimental class delivered essays with rich content closely correlated to the topic based on prior knowledge and writing skill construction.

Problem-based writing instruction was an effective strategy for teaching argumentative writing skill particularly. Compared with guided-writing instruction, problem-based writing instruction had a significant influence on organization, vocabulary, and grammar (Jumariati and Sulistyo, 2017). However, in this study, students from the experimental class shared a slight advantage in grammar. Grammar of post-writing between the two classes did not show significant differences, which is consistent with a previous study that showed that students in content and language integrated learning (CLIL) context performed higher than formal instruction on grammar in their writing but with no significant difference (Vidal and Roquet, 2015). Although much time is spent on grammar instruction in writing teaching, EFL students do not make significant progress on grammar (Huang and Zhang, 2019).

In terms of argumentative structure, the analysis found significant difference in the post-writing between the two classes. The results indicated that the PADE model, which included collaborative teaching activity and knowledge construction, was effective on students’ individually written text (Wigglesworth and Storch, 2012). Teachers introduced six elements of argument and their relationship to each other proposed by Toulmin (1958) to offer students conceptual knowledge of argumentative structure. Osborne et al. (2004) argumentation assessment rubric was offered to the students to find out their problems in argumentation and further evaluate each other’s argumentative writing quality. During the collaboration of the PADE class, students learned to organize their ideas under the argumentative structure. Noticeably, people usually provide more reasons to support their own position in arguments for or against their own positions (Stein and Bernas, 1999), but have difficulties in generating rebuttals. With the help of discussion and exercise session of the PADE model, students learnt to manage conflicts in their oral communication and transfer their critique into argumentative writing. In this study, the experimental class students’ argumentative structure of the second essay was above the middle of levels 3 and 4 after the PADE treatment, which meant that they had a better grasp of presenting claims with data, warrants, and backings in argumentation and some of them even included a claim with a clearly identifiable rebuttal in writing to make a more convincing argumentation, which was the weakest part in constructing argumentation. Students in the comparison class seldom used rebuttals in a persuasive essay with traditional English writing teaching.

### Students’ Perception of the Presentation-Assimilation-Discussion-Exercise Model

Questionnaires were essential to learn students’ attitudes toward the PADE teaching model. After the PADE treatment, students generally showed good acceptability of the PAD teaching in argumentation. For the scale of the PADE course design, students were mostly satisfied with presentation session. Besides, they agreed that the PADE teaching created a free and relaxed teaching environment that facilitated students’ interactive learning in class and after class. Group members exchanged and shared their ideas freely to promote their understanding and memorization of the content they learned. In general, students showed positive attitudes toward the PADE’s course design. As for the teaching effect of the PADE, timely feedback and summary were mostly in favor of, followed by writing competence improvement. Peer feedback works as an effective way to improve learning in L2 writing as a collaborative activity (Yu and Lee, 2016). From students’ perspective, students were improved mostly by the active learning environment. The PADE teaching model had promoted students’ effective learning within groups and class, and fostered their interest to learn English. Most students admitted that they enhanced their problem-solving skills during the PADE learning. With the help of discussion, common problems among students were easily found and promptly solved. Students initiate more problems and are more aware of their weakness (Li and Vandermensbrugghe, 2011). At the same time, students’ communication skill was gradually improved. In the PADE study, students learned to study by themselves, which was useful in their later study of other disciplines. After receiving the PADE teaching of English writing, nearly two-thirds of students were supportive of the PADE teaching model and were in favor of the use of the PADE teaching in other courses in the future. As a consequence, students were generally satisfied with the PADE teaching.

## Conclusion and Limitations

In response to the call to reform the traditional way of English teaching, this study made references to the PAD model and redesigned the PADE model to adapt to the realities of English writing class. The finding of this study suggested that the PADE model had a significant effect on improving students’ vocabulary, content, argumentative structure, and overall quality in argumentative writing. By observing students’ writings, students from the experimental class chose more specific words, complex sentence patterns, and well-structured argumentation with clear argumentative structure. In addition, their argumentative structural level kept rising during the teaching practice. The reason was probably that the PADE class provided students more opportunities to express their opinions, share their understanding on the content and writing topic, and discuss their language and writing problems with group members. In this way, students not only consolidated what they learned, but also improved their English writing competence.

Though the research findings prove the effect of the PADE teaching practice in argumentative writing teaching for college students, it also has some limitations. First, only non-English major participants of intermediate English level took part in the teaching practice, and the research findings might not be definite. In this way, future researchers should include larger scale of participants of various English levels to indicate a more objective and massive map of effect of the PADE model on argumentative writing. Second, this study conducted 14 weeks of argumentative teaching to highlight the effectiveness of the PADE model concerning the requirements to finish the teaching plan and content in one semester. Longer period of writing teaching or teaching practices in English-majored classes might produce more detailed description of students’ writing performance development. In response to technology advancement in the information era, some studies attempted to develop students’ argumentative writing with the help of online tools (Lu and Zhang, 2013). Technological methods could be used to monitor students’ writing progress during the teaching practice in the future. Moreover, this study focused on argumentative writing teaching specifically. Thus, the effect of the PADE model on other writing styles was not analyzed. Future studies could examine the PADE model’s teaching effect on various writing styles to show its influence on different writing styles. Research could also be done to study the impact of the PADE model teaching on college students’ listening, speaking, and reading learning, respectively, in various contexts.

## Data Availability Statement

The original contributions presented in this study are included in the article/supplementary material, further inquiries can be directed to the corresponding author.

## Ethics Statement

The studies involving human participants were reviewed and approved by the Hainan Medical University. Written informed consent for participation was required for this study in accordance with the national legislation and the institutional requirements.

## Author Contributions

ML designed the study and discussed with YL. ML and YL assessed the students’ essays. ML collected the data and wrote the manuscript draft. YL revised the manuscript. Both authors contributed to the manuscript and agreed on the submitted version.

## Conflict of Interest

The authors declare that the research was conducted in the absence of any commercial or financial relationships that could be construed as a potential conflict of interest.

## Publisher’s Note

All claims expressed in this article are solely those of the authors and do not necessarily represent those of their affiliated organizations, or those of the publisher, the editors and the reviewers. Any product that may be evaluated in this article, or claim that may be made by its manufacturer, is not guaranteed or endorsed by the publisher.

## Footnotes

1
www.pigai.org

## Appendix

**TABLE A1:** Modified argumentation rating rubric of Hedgcock and Lefkowitz (1992).

	Score criteria	
Content	76–100	Excellent to very good: knowledgeable; substantive, thorough development of thesis; relevant to topic assigned.
	51–75	Good to average: some knowledge of subject; adequate range; limited thematic development; mostly relevant to topic, but lacks detail.
	26–50	Fair to poor: limited knowledge of subject; minimal substance; poor thematic development.
	0–25	Very poor: shows little or no knowledge of subject; inadequate quantity; not relevant, or not enough to rate.
Organization	76–100	Excellent to very good: fluent expression; clear statement of ideas; solid support; clear organization; logical and cohesive sequencing.
	51–75	Good to average: adequate fluency; main ideas clear but loosely organized; supporting material limited; sequencing logical but incomplete.
	26–50	Fair to poor: low fluency; ideas not well connected; logical sequencing and development lacking.
	0–25	Very poor: ideas not communicated; organization lacking, or not enough to rate.
Grammar	76–100	Excellent to very good: accurate use of relatively complex structures; few errors in agreement, number, tense, word order, articles, pronouns, prepositions.
	51–75	Good to average: simple constructions used effectively; some problems in use of complex constructions; errors in agreement, number, tense, word order, articles, pronouns, prepositions.
	26–50	Fair to poor: significant defects in use of complex constructions; frequent errors in agreement, number, tense, negation, word order, articles, pronouns, prepositions; fragments and deletions; lack of accuracy interferes with meaning.
	0–25	Very poor: no mastery of simple sentence construction; text dominated by errors; does not communicate, or not enough to rate.
Vocabulary	76–100	Excellent to very good: complex range; accurate word/idiom choice; mastery of word forms; appropriate register.
	51–75	Good to average: adequate range; errors of word/idiom choice; effective transmission of meaning.
	26–50	Fair to poor: limited range; frequent word/idiom errors; inappropriate choice, usage; meaning not effectively communicated.
	0–25	Very poor: translation-based errors; little knowledge of target language vocabulary, or not enough to rate.

**TABLE A2:** Analytical framework for assessing the quality of argumentation by Osborne et al. (2004).

Level	
Level 1	Argumentation consists of arguments that are a simple claim versus a counterclaim or a claim versus claim.
Level 2	Argumentation has arguments consisting of claims with either data, warrants, or backings, but do not contain any rebuttals.
Level 3	Argumentation has arguments with a series of claims or counterclaims with either data, warrants, or backings with the occasional weak rebuttal.
Level 4	Argumentation shows arguments with a claim with a clearly identifiable rebuttal. Such an argument may have several claims and counterclaims as well, but this is not necessary.
Level 5	Argumentation displays an extended argument with more than one rebuttal.
